# Supplementary material for: Physics-informed neural networks for modeling physiological time series for cuffless blood pressure estimation
Source: NPJ Digit Med. 2023 Jun 9;6:110. doi: 10.1038/s41746-023-00853-4 (PMC10256762; doi:10.1038/s41746-023-00853-4)
Supplement: Supplementary file 1 — Supplementary Information [file 41746_2023_853_MOESM1_ESM.pdf]

SUPPLEMENTARY INFORMATION

for

Physics-Informed Neural Networks for Modeling Physiological Time Series for Cuffless Blood Pressure Estimation

Kaan Sel[1], Amirmohammad Mohammadi[2], Roderic I. Pettigrew[3] and Roozbeh Jafari[1,2,3*]

[1]Department of Electrical and Computer Engineering, Texas A&M University, College Station, TX, USA

[2]Department of Computer Science and Engineering, Texas A&M University, College Station, TX, USA

[3]School of Engineering Medicine, Texas A&M University, Houston, TX, USA

*corresponding author: rjafari@tamu.edu

Supplementary Table 1

Supplementary Table 1. The BP range and distributions for each participant. The blood pressure categories are defined based on ACC/AHA guidelines[1]. Normal: Less than 120/80 mm Hg; Elevated: Systolic between 120-129 and diastolic less than 80; Stage 1: Systolic between 130-139 or diastolic between 80-89; Stage 2: Systolic at least 140 or diastolic at least 90; Crisis: Systolic over 180 and/or diastolic over 120, all values in mmHg.

Dataset	Subject ID	BP min-max(range) in mmHg		Dataset frequency (in %) for blood pressure categories				
		SBP	DBP	Normal	Elevated	Stage 1	Stage 2	Crisis
Graphene-HGCPT	1	96-168(72)	62-115(52)	20.8	1.1	54.4	40.0	0.0
	2	113-183(70)	71-130(59)	0.4	0.7	40.0	86.0	1.5
	3	116-190(74)	50-97(47)	0.5	14.1	32.8	60.8	2.0
	4	100-163(62)	54-111(56)	49.4	17.5	17.7	21.9	0.0
	5	108-161(53)	53-100(47)	20.6	30.1	36.3	20.1	0.0
	6	114-217(102)	70-140(69)	0.7	3.9	24.9	77.3	3.9
Calfree-HGCPT	7	120-202(82)	55-104(48)	0.0	8.8	49.1	60.4	2.1
	8	100-144(44)	63-105(41)	53.0	17.2	27.4	6.2	0.0
	9	113-193(79)	65-116(50)	0.9	12.3	50.6	56.7	2.6
	10	91-181(90)	52-125(72)	17.5	28.2	19.4	39.1	0.9
	11	116-186(69)	68-113(45)	0.9	10.9	65.8	51.3	1.0
Ring-CPT	12	120-162(41)	61-93(32)	0.0	13.1	66.3	67.6	0.0
	13	84-128(44)	54-79(24)	88.9	11.1	0.0	0.0	0.0
	14	99-166(66)	48-87(38)	16.3	26.5	37.4	42.0	0.0
	15	131-196(64)	83-125(42)	0.0	0.0	13.4	92.1	13.7

Supplementary Table 2

Supplementary Table 2. Graphene-HGCPT dataset. Model performances in SBP and DBP estimation. Values are averaged over all participants for each dataset.

BP estimation model	Training set criterion	% of training labels	Systolic BP		Diastolic BP	
			RMSE, mmHg	Correlation	RMSE,	Correlation
AdaBoost Regressor	Minimal training	7% for SBP, 5% for DBP	13.0	0.55	9.9	0.56
	4-fold cross-validation	~75%	11.0	0.53	8.9	0.48
	8-fold cross-validation	~88%	10.8	0.54	8.7	0.52
Rocket Regressor	Minimal training	7% for SBP, 5% for DBP	12.0	0.60	10.3	0.55
	4-fold cross-validation	~75%	11.1	0.53	9.3	0.44
	8-fold cross-validation	~88%	10.5	0.58	8.8	0.48
Random Forest Regressor	minimal training	7% for SBP, 5% for DBP	11.6	0.58	8.9	0.59
	4-fold cross-validation	~75%	10.8	0.52	8.6	0.49
	8-fold cross-validation	~88%	10.4	0.56	8.3	0.52
LSTM	minimal training (20% validation reserve)	7% for SBP, 5% for DBP	13.8	0.53	11.9	0.49
CNN+Bi-GRU+Attention	minimal training (20% validation reserve)	7% for SBP, 5% for DBP	15.3	0.15	13.2	0.3
ResNet	minimal training (20% validation reserve)	7% for SBP, 5% for DBP	16.8	-0.11	15.2	-0.12
Transformer	minimal training (20% validation reserve)	7% for SBP, 5% for DBP	13.6	0.53	11.7	0.5
CNN (i.e., without $\mathcal{L}_{physics}$)	minimal training (20% validation reserve)	7% for SBP, 5% for DBP	12.5	0.47	10.2	0.53
CNN (i.e., without $\mathcal{L}_{physics}$)	minimal training	7% for SBP, 5% for DBP	14.7	0.57	12.0	0.52
PINN (i.e. with $\mathcal{L}_{physics}$)	minimal training	7% for SBP, 5% for DBP	7.8	0.81	7.1	0.73

Supplementary Table 3

Supplementary Table 3. Calfree-HGCPT dataset. Model performances in SBP and DBP estimation. Values are averaged over all participants for each dataset.

BP estimation model	Training set criterion	% of training labels	Systolic BP		Diastolic BP	
			RMSE, mmHg	Correlation	RMSE,	Correlation
AdaBoost Regressor	Minimal training	5% for SBP,<br>4% for DBP	11.3	0.58	8.5	0.62
	4-fold cross-validation	~75%	10.5	0.55	7.0	0.65
	8-fold cross-validation	~88%	9.9	0.58	6.8	0.66
Rocket Regressor	Minimal training	5% for SBP,<br>4% for DBP	11.7	0.56	8.9	0.60
	4-fold cross-validation	~75%	10.6	0.52	7.4	0.63
	8-fold cross-validation	~88%	10.1	0.56	7.3	0.64
Random Forest Regressor	minimal training	5% for SBP,<br>4% for DBP	11.4	0.54	8.2	0.61
	4-fold cross-validation	~75%	10.4	0.52	7.3	0.61
	8-fold cross-validation	~88%	10.0	0.56	6.9	0.65
LSTM	minimal training<br>(20% validation reserve)	5% for SBP,<br>4% for DBP	14.7	0.45	8.2	0.62
CNN+Bi-GRU+Attention	minimal training<br>(20% validation reserve)	5% for SBP,<br>4% for DBP	15.7	-0.21	11.6	-0.19
ResNet	minimal training<br>(20% validation reserve)	5% for SBP,<br>4% for DBP	14.4	-0.14	11.8	-0.34
Transformer	minimal training<br>(20% validation reserve)	5% for SBP,<br>4% for DBP	13.8	0.46	8.4	0.62
CNN<br>(i.e., without $\mathcal{L}_{physics}$)	minimal training<br>(20% validation reserve)	5% for SBP,<br>4% for DBP	12.7	0.53	8.4	0.66
CNN<br>(i.e., without $\mathcal{L}_{physics}$)	minimal training	5% for SBP,<br>4% for DBP	13.9	0.53	8.7	0.65
PINN<br>(i.e. with $\mathcal{L}_{physics}$)	minimal training	5% for SBP,<br>4% for DBP	8.1	0.77	6.1	0.76

Supplementary Table 4

Supplementary Table 4. Ring-CPT dataset. Model performances in SBP and DBP estimation. Values are averaged over all participants for each dataset.

BP estimation model	Training set criterion	% of training labels	Systolic BP		Diastolic BP	
			RMSE, mmHg	Correlation	RMSE,	Correlation
AdaBoost Regressor	Minimal training	12% for SBP, 7% for DBP	7.2	0.74	5.3	0.69
	4-fold cross-validation	~75%	6.8	0.72	4.9	0.64
	8-fold cross-validation	~88%	6.6	0.75	4.6	0.69
Rocket Regressor	Minimal training	12% for SBP, 7% for DBP	6.8	0.78	5.0	0.72
	4-fold cross-validation	~75%	6.8	0.75	4.5	0.71
	8-fold cross-validation	~88%	6.5	0.78	4.3	0.75
Random Forest Regressor	minimal training	12% for SBP, 7% for DBP	6.7	0.75	4.8	0.71
	4-fold cross-validation	~75%	6.7	0.72	4.8	0.64
	8-fold cross-validation	~88%	6.3	0.75	4.5	0.70
LSTM	minimal training (20% validation reserve)	12% for SBP, 7% for DBP	7.8	0.77	5.1	0.7
CNN+Bi-GRU+Attention	minimal training (20% validation reserve)	12% for SBP, 7% for DBP	17.9	0.05	12.4	-0.11
ResNet	minimal training (20% validation reserve)	12% for SBP, 7% for DBP	18.2	0.03	11.6	0.05
Transformer	minimal training (20% validation reserve)	12% for SBP, 7% for DBP	8.0	0.77	5.2	0.69
CNN (i.e., without $\mathcal{L}_{physics}$)	minimal training (20% validation reserve)	12% for SBP, 7% for DBP	10.7	0.53	5.0	0.71
CNN (i.e., without $\mathcal{L}_{physics}$)	minimal training	12% for SBP, 7% for DBP	8.6	0.76	5.2	0.72
PINN (i.e. with $\mathcal{L}_{physics}$)	minimal training	12% for SBP, 7% for DBP	5.4	0.88	3.8	0.80

Supplementary Table 5

Supplementary Table 5. SBP estimation results for each participant in the graphene-CPT dataset. RMSE: root-mean-squared error, ME: mean error, SDE: standard deviation of the error, R: Pearson's correlation coefficient.

SBP	Graphene-CPT		PINN				CNN			
**Subject ID**	Percent of training instances	Total number of samples	RMSE (mmHg)	ME (mmHg)	SDE (mmHg)	R	RMSE (mmHg)	ME (mmHg)	SDE (mmHg)	R
**1**	3.67	1,878	6.98	0.48	6.97	0.84	10.76	-1.04	10.71	0.73
**2**	3.64	1,867	8.05	2.71	7.58	0.73	15.29	3.09	14.98	0.51
**3**	7.93	920	8.24	3.22	7.59	0.86	11.70	-0.73	11.68	0.77
**4**	8.25	727	7.32	1.17	7.22	0.84	10.92	1.68	10.79	0.70
**5**	8.35	599	8.32	3.96	7.32	0.69	13.23	4.6	12.41	0.32
**6**	7.83	1,060	8.04	0.6	8.02	0.88	26.29	7.62	25.16	0.37
**Average**	6.61	1,175	7.82	2.02	7.45	0.81	14.70	2.54	14.29	0.57

Supplementary Table 6

Supplementary Table 6. SBP estimation results for each participant in the calfree-HGCPT dataset. RMSE: root-mean-squared error, ME: mean error, SDE: standard deviation of the error, R: Pearson's correlation coefficient.

SBP	Calfree-HGCPT		PINN				CNN			
**Subject ID**	Percent of training instances	Total number of samples	RMSE (mmHg)	ME (mmHg)	SDE (mmHg)	R	RMSE (mmHg)	ME (mmHg)	SDE (mmHg)	R
**7**	4.83	1,574	8.22	2.98	7.66	0.82	18.30	1.06	18.27	0.40
**8**	3.52	1,278	7.39	2.27	7.03	0.59	10.77	2.77	10.41	0.39
**9**	5.37	1,453	8.73	0.8	8.70	0.80	16.12	2.91	15.85	0.53
**10**	6.47	1,237	8.49	1.58	8.34	0.85	12.98	2.6	12.72	0.74
**11**	7.12	955	7.45	0.63	7.42	0.78	11.29	2.1	11.09	0.60
**Average**	5.46	1,299	8.06	1.65	7.83	0.77	13.89	2.29	13.67	0.53

Supplementary Table 7

Supplementary Table 7. SBP estimation results for each participant in the ring-CPT dataset. RMSE: root-mean-squared error, ME: mean error, SDE: standard deviation of the error, R: Pearson's correlation coefficient.

SBP	Ring-CPT		PINN				CNN			
**Subject ID**	Percent of training instances	Total number of samples	RMSE (mmHg)	ME (mmHg)	SDE (mmHg)	R	RMSE (mmHg)	ME (mmHg)	SDE (mm Hg)	R
**12**	8.32	469	5.17	0.2	5.16	0.83	8.66	0.2	8.66	0.69
**13**	7.41	607	5.08	-0.89	5.00	0.82	7.91	-1.23	7.81	0.64
**14**	24.08	245	5.88	0.1	5.88	0.92	9.34	1.22	9.26	0.80
**15**	7.40	878	5.48	-0.32	5.47	0.95	8.50	-0.28	8.50	0.89
**Average**	11.80	550	5.40	-0.23	5.38	0.88	8.60	-0.02	8.56	0.76

Supplementary Table 8

Supplementary Table 8. DBP estimation results for each participant in the graphene-CPT dataset. RMSE: root-mean-squared error, ME: mean error, SDE: standard deviation of the error, R: Pearson's correlation coefficient.

DBP	Graphene-CPT		PINN				CNN			
**Subject ID**	Percent of training instances	Total number of samples	RMSE (mmHg)	ME (mmHg)	SDE (mmHg)	R	RMSE (mmHg)	ME (mmHg)	SDE (mmHg)	R
**1**	2.77	1,878	6.64	-0.81	6.59	0.77	8.79	-1.44	8.67	0.67
**2**	3.05	1,867	7.62	1.83	7.40	0.65	13.55	-1.39	13.48	0.46
**3**	4.78	920	6.51	2.47	6.03	0.72	9.75	3.53	9.09	0.61
**4**	7.70	727	6.52	0.06	6.52	0.85	9.61	1.8	9.44	0.71
**5**	7.68	599	6.38	1.23	6.26	0.77	12.59	-1.06	12.55	0.40
**6**	6.13	1,060	9.01	-0.35	9.00	0.63	17.69	2.64	17.49	0.27
**Average**	5.35	1,175	7.11	0.74	6.97	0.73	12.00	0.68	11.79	0.52

Supplementary Table 9

Supplementary Table 9. DBP estimation results for each participant in the calfree-HGCPT dataset. RMSE: root-mean-squared error, ME: mean error, SDE: standard deviation of the error, R: Pearson's correlation coefficient.

DBP	Calfree-HGCPT		PINN				CNN			
**Subject ID**	Percent of training instances	Total number of samples	RMSE (mmHg)	ME (mmHg)	SDE (mmHg)	R	RMSE (mmHg)	ME (mmHg)	SDE (mmHg)	R
**7**	3.11	1,574	5.82	1.11	5.71	0.82	8.20	1.2	8.11	0.71
**8**	3.05	1,278	5.93	2.88	5.18	0.60	8.04	3.07	7.43	0.51
**9**	3.44	1,453	7.01	1.83	6.77	0.80	8.44	0.92	8.39	0.74
**10**	5.74	1,237	6.17	-0.2	6.16	0.91	10.77	-1.47	10.67	0.77
**11**	4.29	955	5.62	1.07	5.52	0.66	7.87	1.32	7.76	0.51
**Average**	3.93	1,299	6.11	1.34	5.87	0.76	8.66	1.01	8.47	0.65

Supplementary Table 10

Supplementary Table 10. DBP estimation results for each participant in the ring-CPT dataset. RMSE: root-mean-squared error, ME: mean error, SDE: standard deviation of the error, R: Pearson's correlation coefficient.

DBP	Ring-CPT		PINN				CNN			
**Subject ID**	Percent of training instances	Total number of samples	RMSE (mmHg)	ME (mmHg)	SDE (mmHg)	R	RMSE (mmHg)	ME (mmHg)	SDE (mmHg)	R
**12**	6.18	469	4.54	0.47	4.51	0.63	6.71	-0.12	6.71	0.45
**13**	4.12	607	3.31	-0.03	3.31	0.73	3.73	0.65	3.68	0.73
**14**	14.29	245	3.31	-0.67	3.24	0.92	4.84	-1.18	4.70	0.84
**15**	4.67	878	4.08	-0.84	3.99	0.92	5.40	-0.26	5.39	0.85
**Average**	7.32	550	3.81	-0.27	3.76	0.80	5.17	-0.23	5.12	0.72

Supplementary Table 11

Supplementary Table 11. PP estimation results for each participant in the graphene-CPT dataset. RMSE: root-mean-squared error, ME: mean error, SDE: standard deviation of the error, R: Pearson's correlation coefficient.

PP	Graphene-CPT		PINN				CNN			
**Subject ID**	Percent of training instances	Total number of samples	RMSE (mmHg)	ME (mmHg)	SDE (mmHg)	R	RMSE (mmHg)	ME (mmHg)	SDE (mmHg)	R
**1**	1.54	1,878	3.65	1.53	3.31	0.74	6.53	0.18	6.53	0.33
**2**	2.57	1,867	10.56	5.99	8.70	0.39	19.63	7.34	18.21	0.10
**3**	6.20	920	6.11	2.99	5.33	0.86	9.74	2.2	9.49	0.70
**4**	3.85	727	4.58	-1.31	4.39	0.59	8.14	-2.88	7.61	0.20
**5**	5.84	599	7.69	3.1	7.04	0.61	16.44	-0.75	16.43	0.04
**6**	3.87	1,060	6.64	2.05	6.31	0.65	10.16	2.27	9.91	0.40
**Average**	3.98	1,175	6.54	2.39	5.85	0.64	11.77	1.39	11.36	0.30

Supplementary Table 12

Supplementary Table 12. PP estimation results for each participant in the calfree-HGCPT dataset. RMSE: root-mean-squared error, ME: mean error, SDE: standard deviation of the error, R: Pearson's correlation coefficient.

PP	Calfree-HGCPT		PINN				CNN			
**Subject ID**	Percent of training instances	Total number of samples	RMSE (mmHg)	ME (mmHg)	SDE (mmHg)	R	RMSE (mmHg)	ME (mmHg)	SDE (mmHg)	R
**7**	3.18	1,574	7.78	2.97	7.19	0.72	16.58	4.19	16.04	0.23
**8**	1.80	1,278	4.48	2.04	3.99	0.43	5.65	2.15	5.23	0.23
**9**	2.55	1,453	5.97	0.97	5.89	0.45	7.59	2.14	7.28	0.39
**10**	2.51	1,237	4.87	2.23	4.33	0.60	6.18	1.35	6.03	0.33
**11**	4.71	955	6.62	1.84	6.36	0.68	11.35	4.46	10.44	0.29
**Average**	2.95	1,299	5.94	2.01	5.55	0.58	9.47	2.86	9.00	0.29

Supplementary Table 13

Supplementary Table 13. PP estimation results for each participant in the ring-CPT dataset. RMSE: root-mean-squared error, ME: mean error, SDE: standard deviation of the error, R: Pearson's correlation coefficient.

PP	Ring-CPT		PINN				CNN			
**Subject ID**	Percent of training instances	Total number of samples	RMSE (mmHg)	ME (mmHg)	SDE (mmHg)	R	RMSE (mmHg)	ME (mmHg)	SDE (mmHg)	R
**12**	4.69	469	3.21	-0.48	3.17	0.81	4.30	0.01	4.30	0.71
**13**	3.62	607	2.95	0.31	2.94	0.75	3.51	0.8	3.42	0.71
**14**	16.33	245	7.72	2.68	7.24	0.66	9.34	4.4	8.23	0.48
**15**	4.10	878	3.52	0.34	3.50	0.89	5.62	0.87	5.55	0.77
**Average**	7.18	550	4.35	0.71	4.21	0.78	5.69	1.52	5.38	0.67

Supplementary Table 14

Supplementary Table 14. AAMI results for SBP estimation. ME: mean error, SDE: standard deviation of error.

	PINN		CNN	
**SBP**	ME (mmHg)	SDE (mmHg)	ME (mmHg)	SDE (mmHg)
AAMI standard	< 5	< 8	< 5	< 8
**Dataset**	ME (mmHg)	SDE (mmHg)	ME (mmHg)	SDE (mmHg)
Calfree-HGCPT	1.75	7.94	2.26	14.42
Graphene-CPT	1.81	7.54	2.15	15.27
Ring-CPT	-0.33	5.33	-0.30	8.45

Supplementary Table 15

Supplementary Table 15. AAMI results for DBP estimation. ME: mean error, SDE: standard deviation of error.

	PINN		CNN	
**DBP**	ME (mmHg)	SDE (mmHg)	ME (mmHg)	SDE (mmHg)
AAMI standard	< 5	< 8	< 5	< 8
**Dataset**	ME (mmHg)	SDE (mmHg)	ME (mmHg)	SDE (mmHg)
Calfree-HGCPT	1.37	6.01	1.03	8.65
Graphene-CPT	0.65	7.23	0.18	12.29
Ring-CPT	-0.32	3.90	-0.07	5.26

Supplementary Table 16

Supplementary Table 16. AAMI results for PP estimation. ME: mean error, SDE: standard deviation of error.

	PINN		CNN	
**PP**	ME (mmHg)	SDE (mmHg)	ME (mmHg)	SDE (mmHg)
AAMI standard	< 5	< 8	< 5	< 8
**Dataset**	ME (mmHg)	SDE (mmHg)	ME (mmHg)	SDE (mmHg)
Calfree-HGCPT	2.03	5.77	2.82	10.18
Graphene-CPT	2.82	6.61	2.30	12.80
Ring-CPT	0.38	3.93	1.01	5.27

Supplementary Table 17

Supplementary Table 17. Out-of-distribution test analysis results. Comparison of blood pressure (BP) estimation model accuracies. Values are averaged over all participants (*N*=15).

**BP estimation model**	**Systolic BP, mmHg**		**Diastolic BP, mmHg**	
	**Mean difference**	**Standard deviation**	**Mean difference**	**Standard deviation**
AdaBoost Regressor	2.3	14.5	2.5	10.6
Rocket Regressor	2.0	10.4	1.9	8.1
Random Forest Regressor	1.7	10.7	2.0	8.0
CNN (i.e., without $\mathcal{L}_{physics}$)	-1.8	12.6	-0.6	8.9
PINN (i.e. with $\mathcal{L}_{physics}$)	-1.3	7.0	-0.7	5.8

Supplementary Table 18

Supplementary Table 18. Leave-one-subject-out analysis. Comparison of blood pressure (BP) estimation model accuracies. Values are averaged over all participants (*N*=15).

**BP estimation model**	**Systolic BP, mmHg**		**Diastolic BP, mmHg**	
	**Mean difference**	**Standard deviation**	**Mean difference**	**Standard deviation**
AdaBoost Regressor	-6.1	16	-3.8	10.2
Rocket Regressor	-2.3	15.3	-0.6	9.5
Random Forest Regressor	-6.7	14.7	-3.8	9.5
CNN (i.e., without $\mathcal{L}_{physics}$)	-2.4	17	1.2	11.6
PINN (i.e. with $\mathcal{L}_{physics}$)	0.2	12.1	1.6	8.7

Supplementary Table 19

Supplementary Table 19. SBP estimation results with PINN models with varying physics-based loss functions: 1st order Taylor series, 2nd order Taylor series, ablation study. The ablation study corresponds to the replacement of physiological features with the first three dimensions of the flatten-layer output in Taylor's approximation.

Dataset	Subject ID	PINN (1st order Taylor)		PINN (2nd order Taylor)		PINN (ablation study)	
		RMSE (mmHg)	Correlation	RMSE (mmHg)	Correlation	RMSE (mmHg)	Correlation
Graphene-HGCPT	1	7.0	0.84	6.9	0.84	8.3	0.79
	2	8.1	0.73	7.8	0.73	9.6	0.65
	3	8.2	0.86	8.1	0.86	18.0	0.64
	4	7.3	0.84	7.1	0.85	failed to converge	
	5	8.3	0.69	7.7	0.74	8.9	0.66
	6	8.0	0.88	7.7	0.90	44.1	0.34
	**Average**	**7.8**	**0.81**	**7.5**	**0.82**	**17.8**	**0.62**
Calfree-HGCPT	7	8.2	0.82	8.2	0.82	17.3	0.55
	8	7.4	0.59	7.3	0.58	10.5	0.41
	9	8.7	0.80	8.4	0.81	18.2	0.41
	10	8.5	0.85	8.3	0.87	failed to converge	
	11	7.4	0.78	7.6	0.77	10.7	0.63
	**Average**	**8.1**	**0.77**	**8.0**	**0.77**	**14.2**	**0.50**
Ring-CPT	12	5.2	0.83	5.3	0.82	12.4	0.47
	13	5.1	0.82	4.6	0.85	7.1	0.75
	14	5.9	0.92	6.3	0.91	10.1	0.72
	15	5.5	0.95	5.1	0.95	7.5	0.89
	**Average**	**5.4**	**0.88**	**5.3**	**0.88**	**9.3**	**0.71**

Supplementary Table 20

Supplementary Table 20. DBP estimation results with PINN models with varying physics-based loss functions: 1st order Taylor series, 2nd order Taylor series, ablation study. The ablation study corresponds to the replacement of physiological features with the first three dimensions of the flatten-layer output in Taylor's approximation.

Dataset	Subject ID	PINN (1st order Taylor)		PINN (2nd order Taylor)		PINN (ablation study)	
		RMSE (mmHg)	Correlation	RMSE (mmHg)	Correlation	RMSE (mmHg)	Correlation
Graphene-HGCPT	1	6.6	0.77	6.6	0.77	8.5	0.63
	2	7.6	0.65	7.5	0.65	7.7	0.58
	3	6.5	0.72	6.6	0.71	6.2	0.71
	4	6.5	0.85	6.7	0.84	failed to converge	
	5	6.4	0.77	6.4	0.77	8.0	0.71
	6	9.0	0.63	8.7	0.65	11.4	0.56
	**Average**	7.1	0.73	7.1	0.73	8.4	0.64
Calfree-HGCPT	7	5.8	0.82	5.7	0.82	6.1	0.76
	8	5.9	0.60	6.1	0.59	10.4	0.25
	9	7.0	0.80	6.7	0.81	28.5	0.09
	10	6.2	0.91	5.8	0.93	14.5	0.66
	11	5.6	0.66	5.3	0.68	45.1	0.05
	**Average**	6.1	0.76	5.9	0.77	20.9	0.36
Ring-CPT	12	4.5	0.63	4.3	0.66	6.7	0.57
	13	3.3	0.73	3.2	0.75	3.4	0.71
	14	3.3	0.92	3.2	0.92	5.2	0.76
	15	4.1	0.92	3.9	0.92	7.5	0.72
	**Average**	3.8	0.80	3.7	0.81	5.7	0.69

Supplementary Table 21

Supplementary Table 21. PP estimation results with PINN models with varying physics-based loss functions: 1st order Taylor series, 2nd order Taylor series, ablation study. The ablation study corresponds to the replacement of physiological features with the first three dimensions of the flatten-layer output in Taylor's approximation.

Dataset	Subject ID	PINN (1st order Taylor)		PINN (2nd order Taylor)		PINN (ablation study)	
		RMSE (mmHg)	Correlation	RMSE (mmHg)	Correlation	RMSE (mmHg)	Correlation
Graphene-HGCPT	1	3.6	0.74	3.7	0.72	3.7	0.67
	2	10.6	0.39	10.7	0.41	11.6	0.20
	3	6.1	0.86	6.1	0.86	9.1	0.72
	4	4.6	0.59	4.6	0.56	5.0	0.65
	5	7.7	0.61	7.3	0.63	7.9	0.66
	6	6.6	0.65	6.5	0.65	failed to converge	
	**Average**	**6.5**	**0.64**	**6.5**	**0.64**	**7.5**	**0.58**
Calfree-HGCPT	7	7.8	0.72	8.3	0.70	11.7	0.49
	8	4.5	0.43	4.4	0.48	4.3	0.50
	9	6.0	0.45	5.9	0.45	13.4	0.34
	10	4.9	0.60	4.7	0.60	4.3	0.69
	11	6.6	0.68	6.7	0.69	11.8	0.41
	**Average**	**5.9**	**0.57**	**6.0**	**0.58**	**9.1**	**0.49**
Ring-CPT	12	3.2	0.81	3.1	0.83	failed to converge	
	13	3.0	0.75	2.9	0.76	3.3	0.68
	14	7.7	0.66	7.0	0.65	48.7	0.37
	15	3.5	0.89	3.4	0.89	37.6	-0.28
	**Average**	**4.4**	**0.78**	**4.1**	**0.78**	**29.9**	**0.26**

Supplementary Figure 1

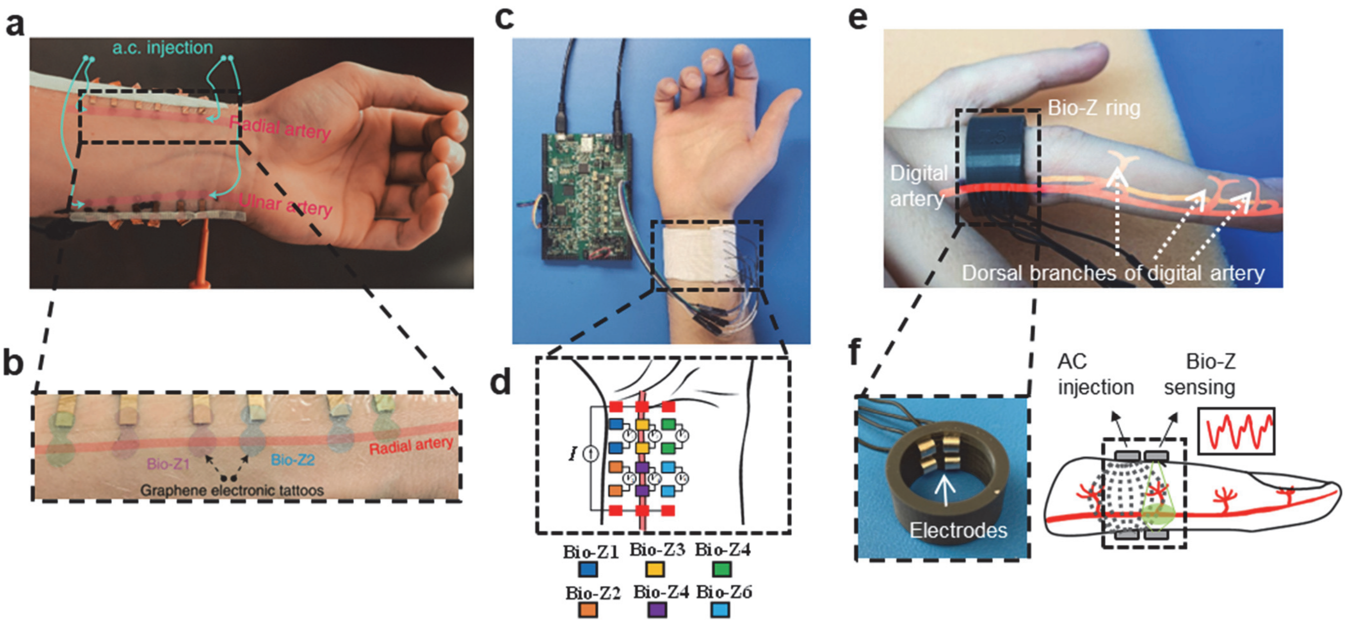

Supplementary Figure 1. Sensors used for bioimpedance signal acquisition. The bioimpedance sensing requires the injection of a high-frequency electric signal via a pair of electrodes building electrical contact with the skin, where a separate pair of electrodes are used to obtain the bioimpedance signal induced by this injected signal. The bioimpedance signal provides measurements of deep tissue characteristics of the human body: tissue and cell compositions and their transient behavior due to physio-mechanical activities (e.g., blood flow, respiration, body fluid shifts, body fat-muscle composition changes). When the bioimpedance sensors are placed along the arteries (e.g., radial artery on wrist, digital artery on finger), the acquired signal changes quasi-periodically with the arterial volume changes due to the arrival of the pulse wave at each heartbeat cycle. We used three bioimpedance datasets, where each dataset includes measurements obtained via bioimpedance sensors consisting of unique electrode types, capturing blood volumetric changes at participants' wrists or fingers. **(a-b)** In the graphene-HGCPT dataset, the bioimpedance sensor electrodes are based on atomically thin, self-adhesive, lightweight and unobtrusive graphene electronic tattoos (GETs)[2]. A total of 12 GETs with a surface area of 25 mm^2 are placed at the participants' wrists aligned with radial and ulnar arteries. In this work, we used bioimpedance signal obtained with electrodes marked as Bio-Z2 (blue color in subplot b). Reproduced with permission[2]. Copyright © 2022, Springer Nature. **(c-d)** In the calfree-HGCPT dataset, a wrist-worn array band consisting of 6 x 8 silver electrodes are used for bioimpedance signal acquisition[3]. Each electrode sized 5 mm x 5 mm, with a 3.2 mm spacing between the electrodes. In this work, we used bioimpedance signal obtained with electrodes marked as Bio-Z3 (yellow color in subplot d). Reproduced with permission[3]. Copyright © 2022, Springer Nature. **(e-f)** In the ring-CPT dataset[4], a ring form-factor bioimpedance sensor consisting of 2 x 2 silver electrodes are used. The rings are easy to wear, not burdensome and provide ideal sensor-to-skin contact. Each participant wore a bioimpedance ring sensor on their ring fingers, where the sensing electrode pair within the sensor is aligned with the underlying digital artery.

Supplementary Figure 2

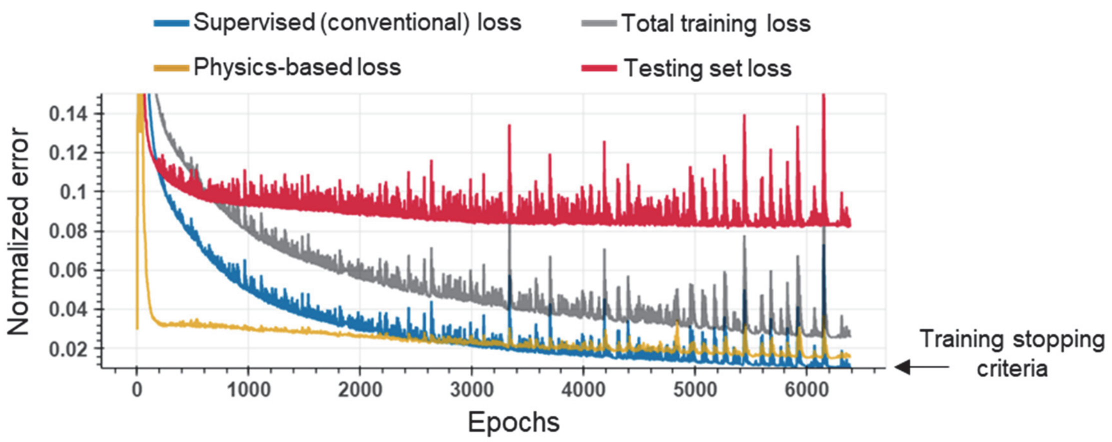

Supplementary Figure 2. Values of the loss functions shown for progressing training epochs for PINN models. The plot is extracted from SID#5's SBP data. Initially, the conventional loss (blue) is an order of magnitude higher than the physics-based loss, dominating the weights optimization. Initially higher values for the supervised loss ensure that the model estimations are bounded by the true range of BP, where eventually, as the supervised loss decreases, the model weight optimization focuses on satisfying the physics-based constraint. In addition, this joint optimization ensures that the model predictions at all times maintain the physics-based constraint, where any physical inconsistency would result in a significant increase in the physics-based loss values.

Supplementary Figure 3

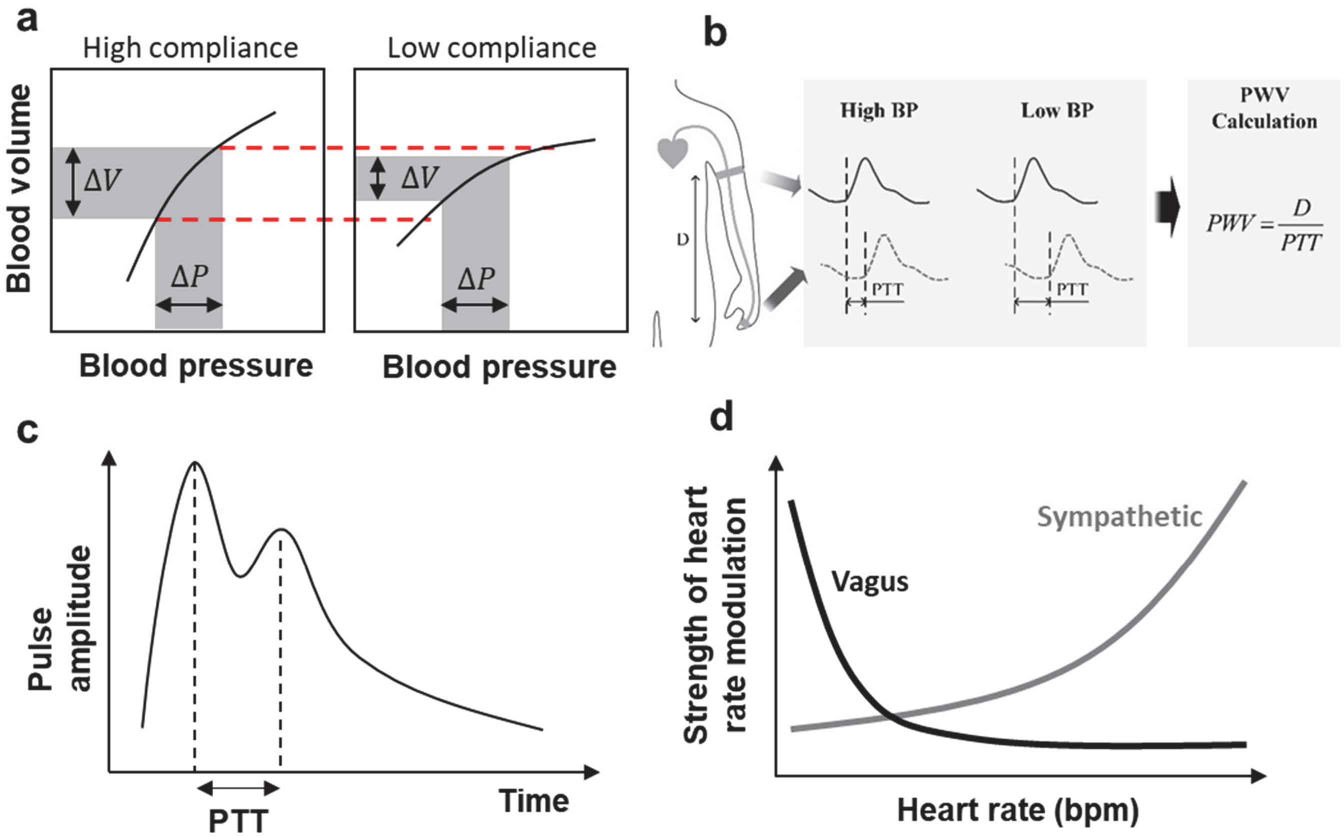

Supplementary Figure 3. Hemodynamic relationships. a. Blood volume – pressure relationship based on different arterial wall characteristics (i.e., varying compliance). An expansion in arterial diameter caused by blood arrival causes an increase in pressure exerted to the arterial wall. The change of pressure w.r.t. volume is dependent on the level of artery compliance[5]. **b.** Pulse wave velocity (PWV), pulse transit time (PTT) and BP relationship. Higher BP corresponds to a higher PWV and decreased PTT. Conventionally, PTT is measured using two (i.e. distal and proximal) points on the arterial tree[6,7]. Reproduced with permission[8]. Copyright © 2022, MDPI. **c.** Pulse wave analysis from wearable sensor measurements to capture a proxy for the PTT-PWV, based on the time difference between the arrivals of the first pressure wave and the reflected pressure wave to the sensing site[9]. **d.** Heart rate response to vagal nerve (e.g., vagal outflow) vs. sympathetic nervous system activities, which causes a variation in the heart rate response to a stressor (e.g., cold-pressor test) that increases blood pressure[10–12].

Supplementary Figure 4

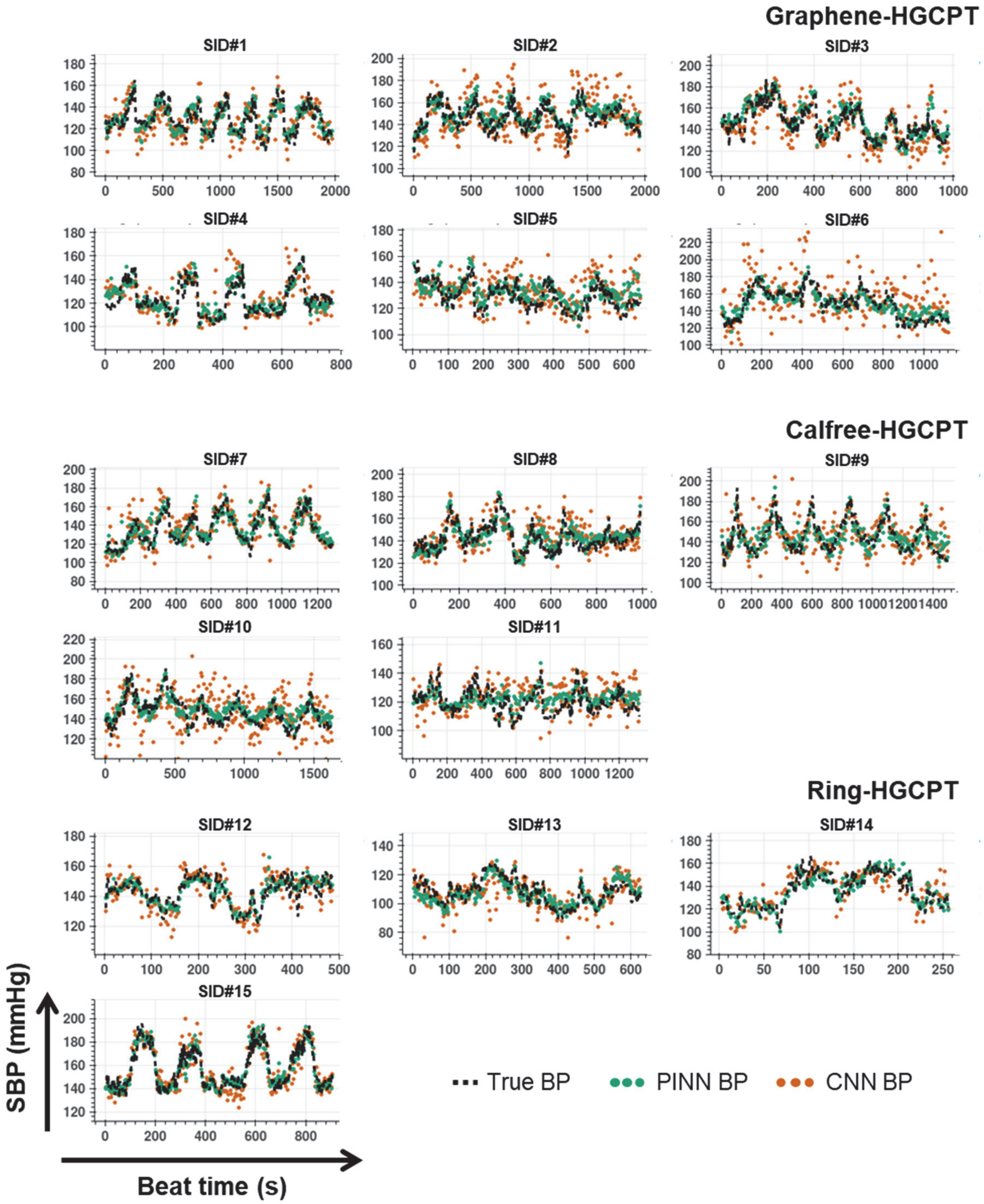

Supplementary Figure 4. Systolic blood pressure (SBP) estimations. The subplots show the estimations for all N=15 participants over three datasets (graphene-HGCPT, calfree-HGCPT, ring-CPT). Scattered points show the model estimations for PINN (green) and CNN (orange) in comparison to ground truth BP shown in dashed line (black). The x-axis of the subplots shows the beat times, and the y-axis shows the SBP in mmHg.

Supplementary Figure 5

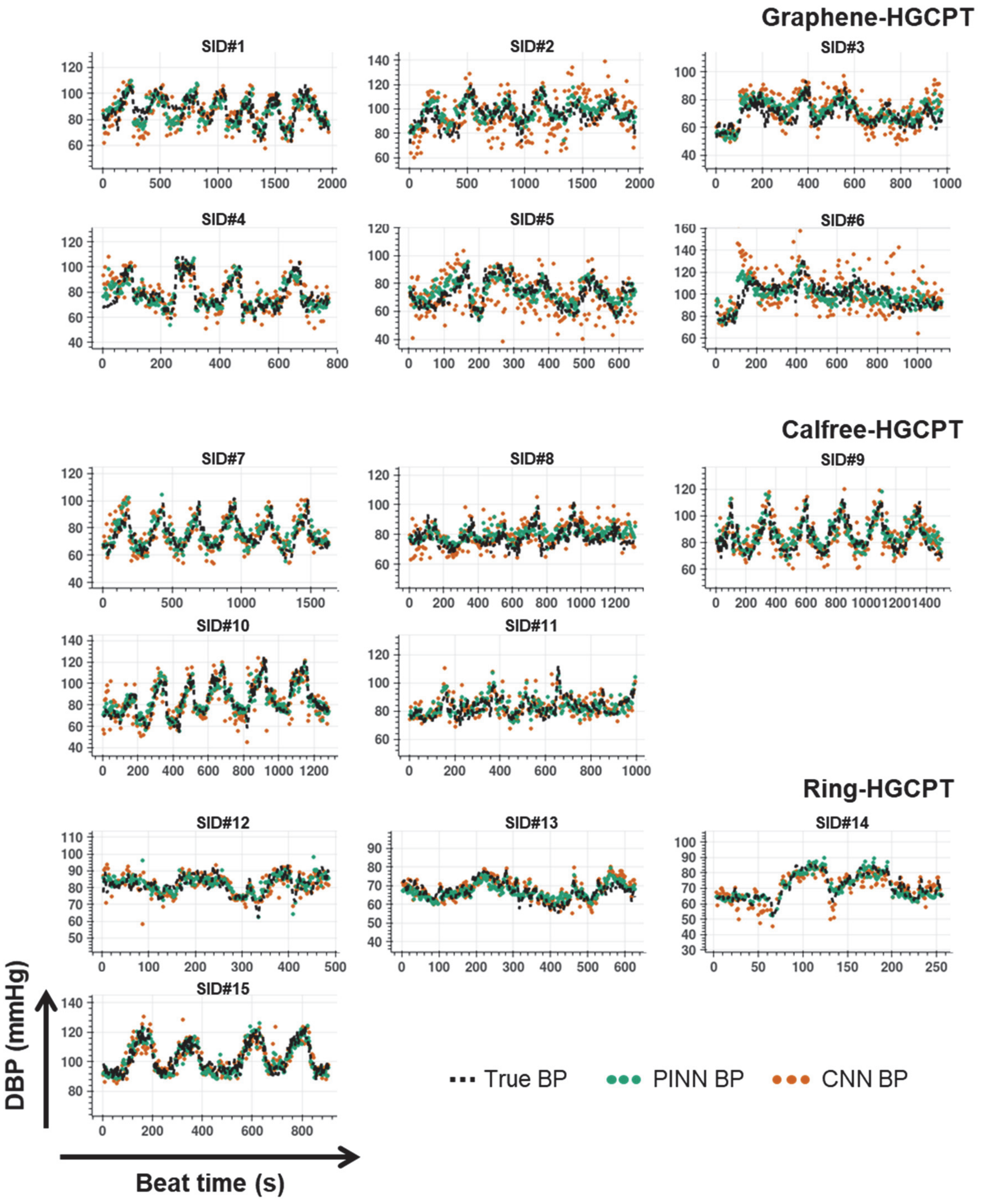

Supplementary Figure 5. Diastolic blood pressure (DBP) estimations. The subplots show the estimations for all N=15 participants over three datasets (graphene-HGCPT, calfree-HGCPT, ring-CPT). Scattered points show the model estimations for PINN (green) and CNN (orange) in comparison to ground truth BP shown in dashed line (black). The x-axis of the subplots shows the beat times, and the y-axis shows the DBP in mmHg.

Supplementary Figure 6

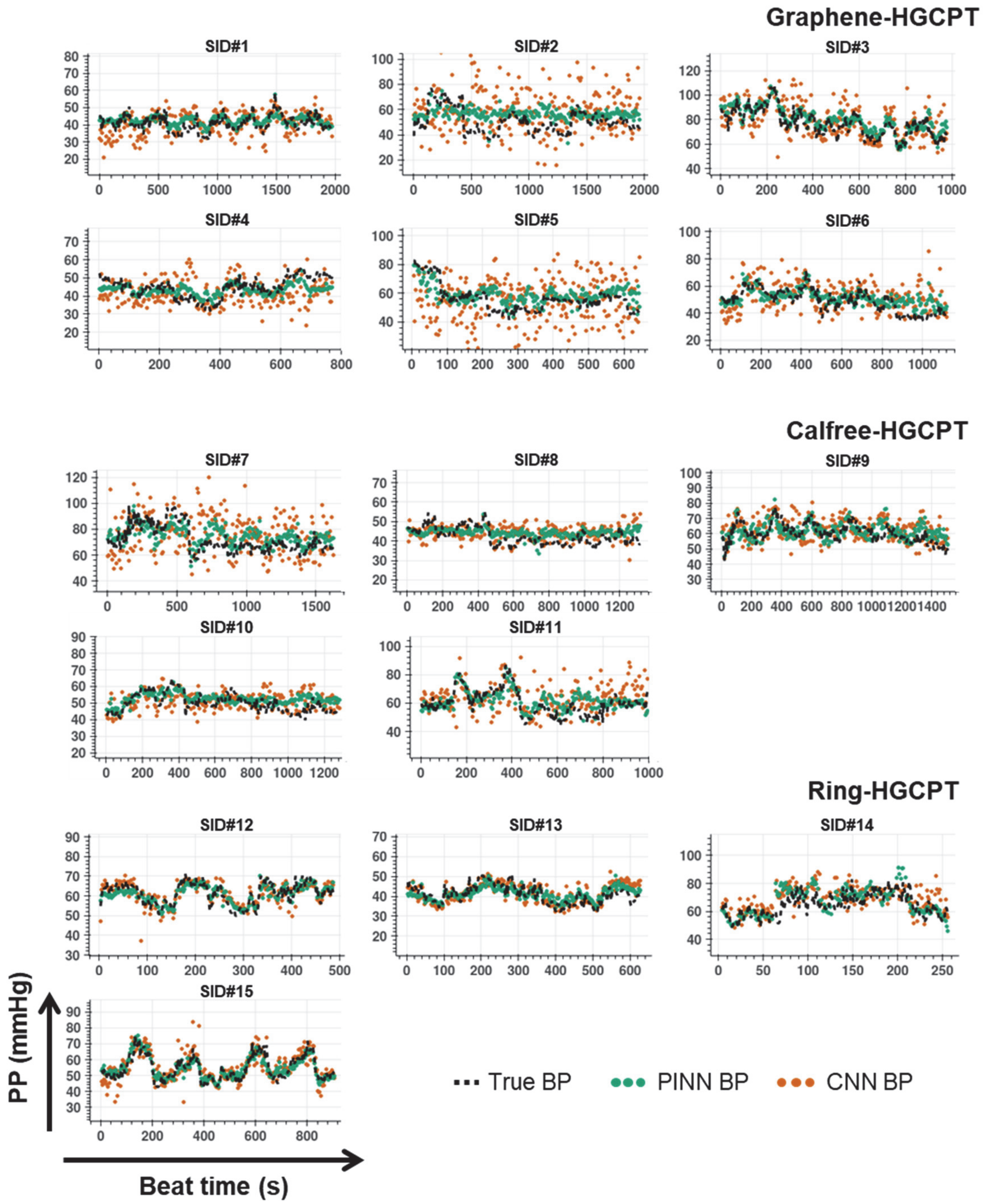

Supplementary Figure 6. Pulse pressure (PP) estimations. The subplots show the estimations for all N=15 participants over three datasets (graphene-HGCPT, calfree-HGCPT, ring-CPT). Scattered points show the model estimations for PINN (green) and CNN (orange) in comparison to ground truth BP shown in dashed line (black). The x-axis of the subplots shows the beat times, and the y-axis shows the PP in mmHg.

Supplementary Figure 7

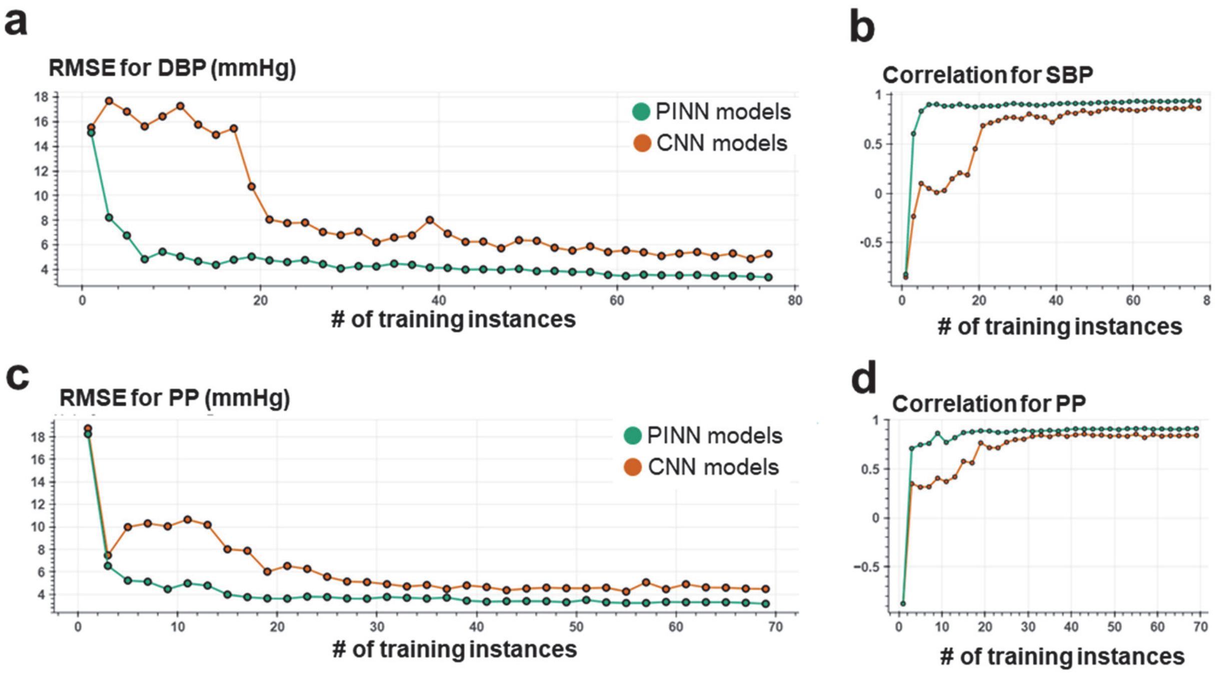

Supplementary Figure 7. PINN and CNN model behavior under increasing number of training instances. (a-b) Root-mean-squared error (RMSE) in mmHg and Pearson's correlation coefficient in estimating DBP for PINN (green) and conventional CNN (orange) models trained with increasing number of training instances. **(c-d)** Root-mean-squared error (RMSE) in mmHg and Pearson's correlation coefficient in estimating PP for PINN (green) and conventional CNN (orange) models trained with increasing number of training instances. PINN models overperforms CNN models independent of the number of labeled instances used for model training. In addition, PINN models show a consistent convergence to a lower error and higher correlation with less than with the gradual increase in the size of the labeled training data.

Supplementary Figure 8

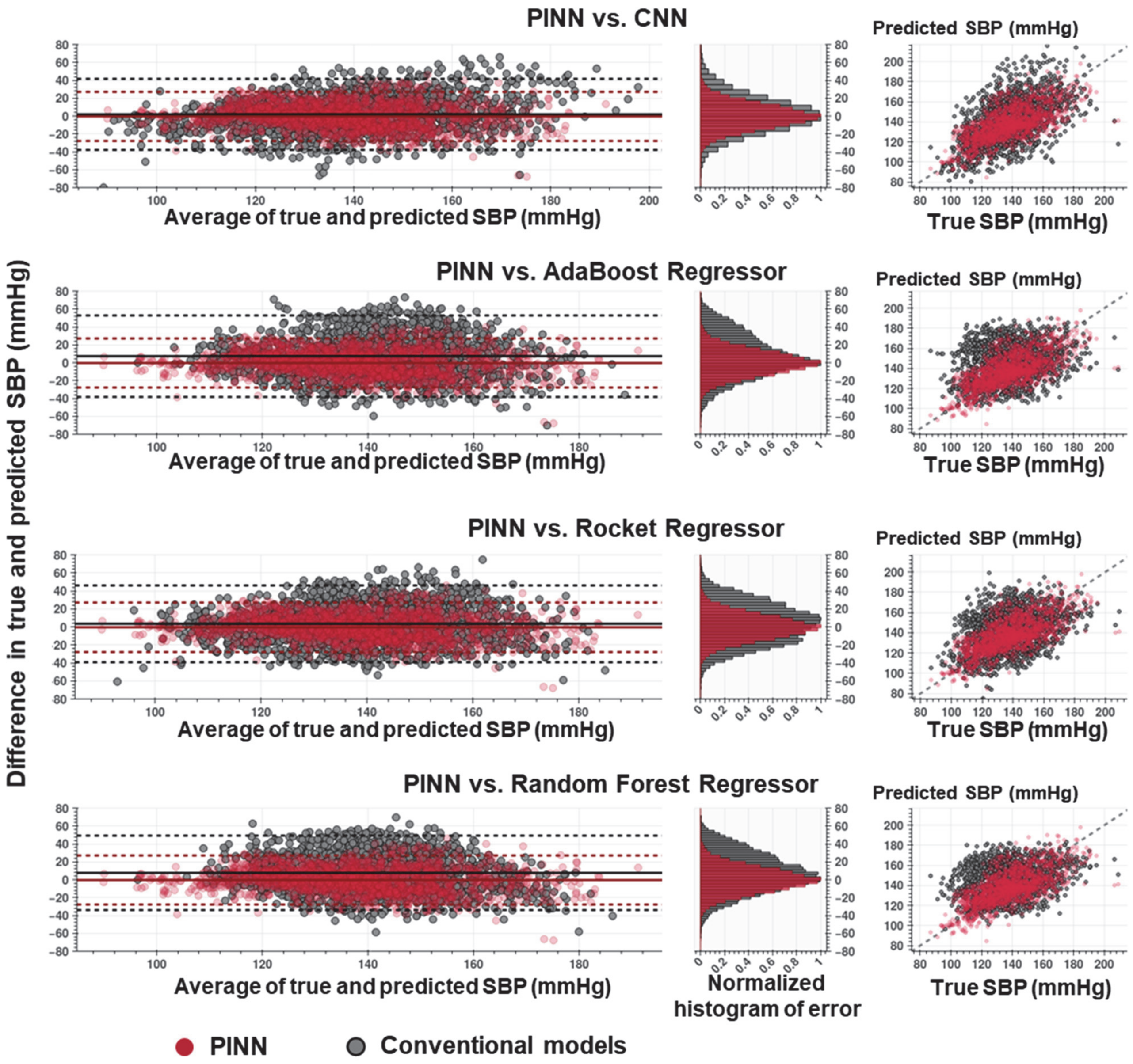

Supplementary Figure 8. Assessment of PINN, CNN, and AdaBoost, Rocket, and Random Forest Regressor SBP estimation performances under inter-subject evaluation strategy (See Supplementary Text 3). The plots are generated using data from all participants ($N = 15$). Red and gray colors show PINN and conventional model accuracies, plotted together for comparison. Left plots present Bland-Altman analysis results. Mean error (ME) ± standard deviations (SD); PINN: 0.3 ± 14.0 mmHg; CNN: -1.9 ± 20.3 mmHg; AdaBoost regressor: -7.2 ± 23.2 mmHg; Rocket regressor: -3.4 ± 21.8 mmHg; Random Forest regressor: -7.6 ± 21.3 mmHg. Middle plots show histograms of SBP and DBP estimation error. Right plots show Pearson's correlation analysis results. Correlation coefficients; PINN: 0.68; CNN: 0.54; AdaBoost regressor: 0.19; Rocket regressor 0.34; Random Forest regressor: 0.21. PINNs show significant improvement in accuracies against all of the tested state-of-the-art models.

Supplementary Figure 9

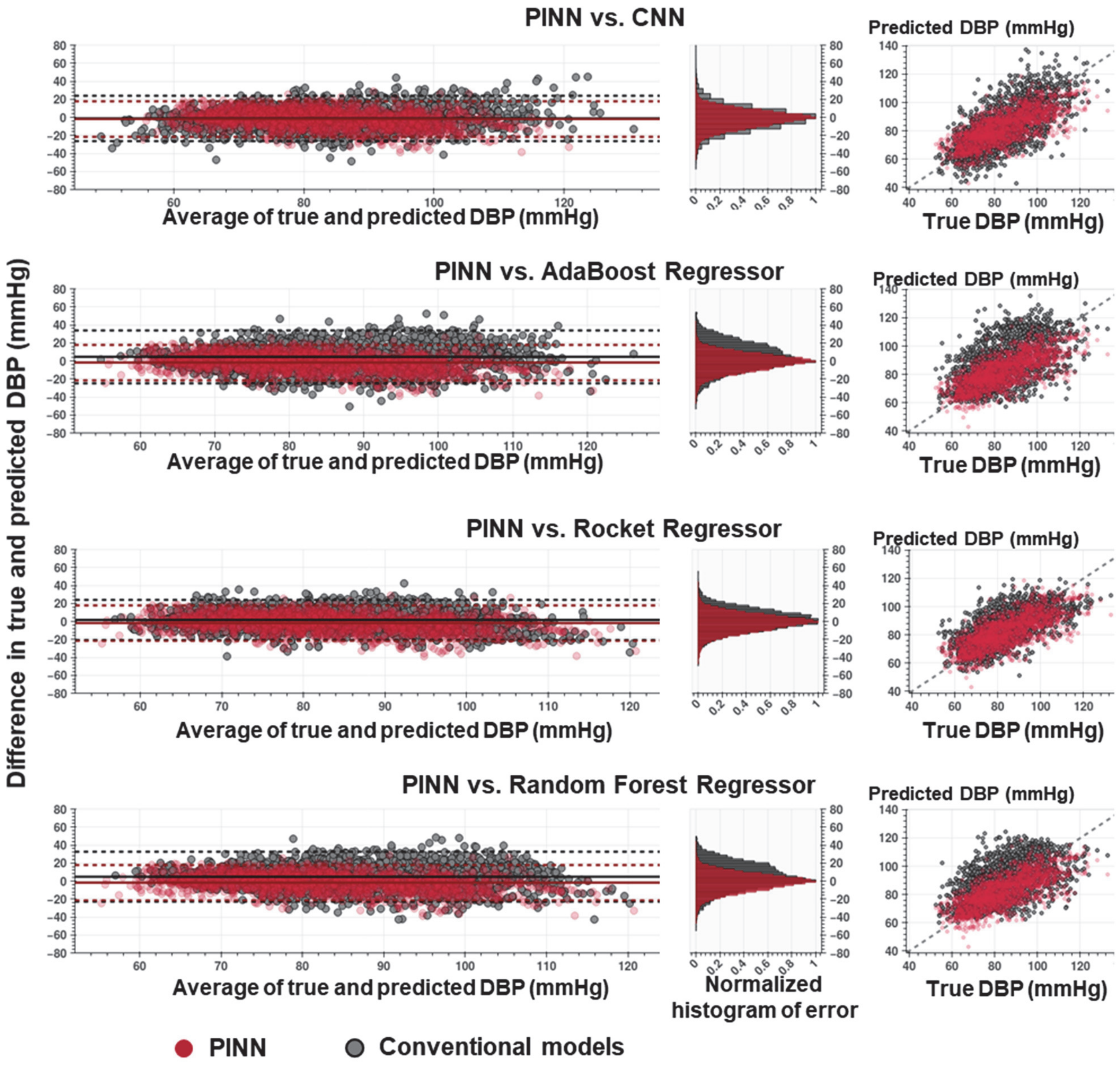

Supplementary Figure 9. Assessment of PINN, CNN, and AdaBoost, Rocket, and Random Forest Regressor DBP estimation performances under inter-subject evaluation strategy (See Supplementary Text 3). The plots are generated using data from all participants ($N = 15$). Red and gray colors show PINN and conventional model accuracies, plotted together for comparison. Left plots present Bland-Altman analysis results. Mean error (ME) ± standard deviations (SD); PINN: 2.0 ± 10.1 mmHg; CNN: -1.2 ± 12.9 mmHg; AdaBoost regressor: -4.5 ± 15.0 mmHg; Rocket regressor: -1.6 ± 11.4 mmHg; Random Forest regressor: -4.8 ± 14.1 mmHg. Middle plots show histograms of SBP and DBP estimation error. Right plots show Pearson's correlation analysis results. Correlation coefficients; PINN: 0.71; CNN: 0.65; AdaBoost regressor: 0.54; Rocket regressor 0.65; Random Forest regressor: 0.52. PINNs show significant improvement in accuracies against all of the tested state-of-the-art models.

Supplementary Figure 10

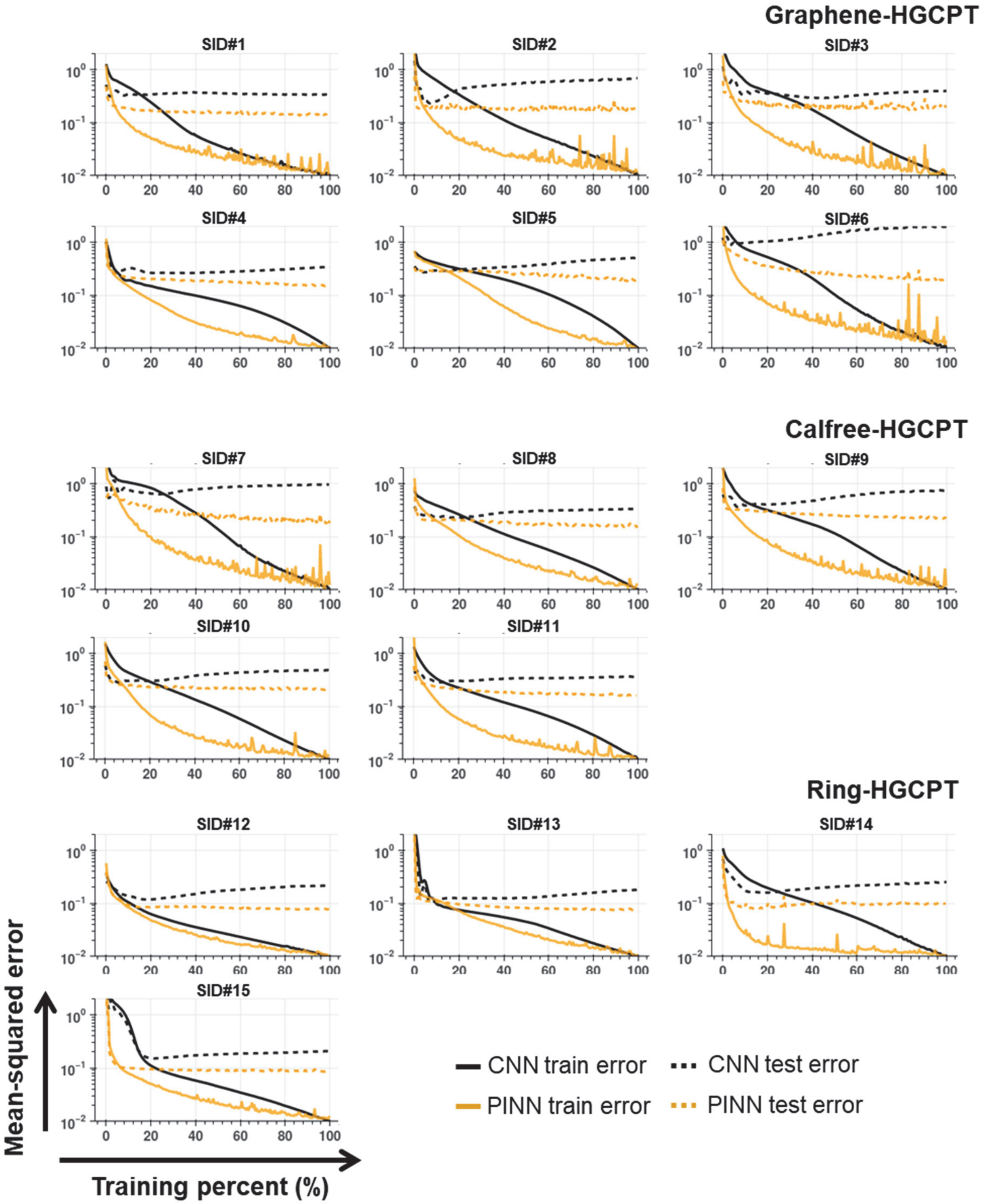

Supplementary Figure 10. PINN and CNN training and testing error progression with training for SBP estimation. For the training errors, only the supervised training losses (i.e., mean-squared different between prediction and true) are shown. The trainings are stopped when the supervised training errors reached 0.01. We observe that the CNN models in almost all cases begin overfitting at some point, where as the train loss decreases, the test loss is increasing. Whereas, for PINN models we observe a converging training loss, where in multiple cases, test loss continues decreasing (e.g. SID#6).

Supplementary Figure 11

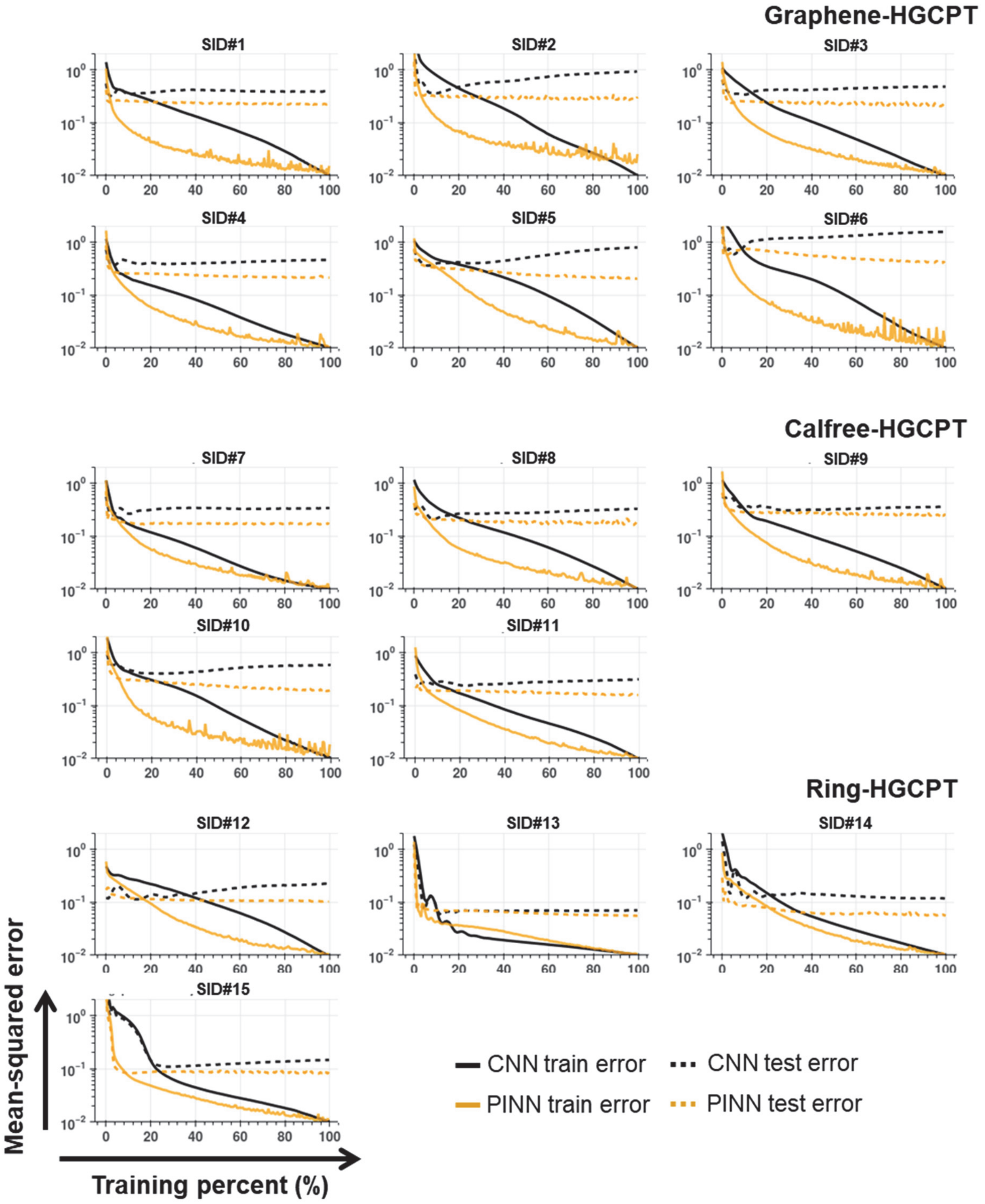

Supplementary Figure 11. PINN and CNN training and testing error progression with training for DBP estimation. For the training errors, only the supervised training losses (i.e., mean-squared different between prediction and true) are shown. The trainings are stopped when the supervised training errors reached 0.01. We observe that the CNN models in almost all cases begin overfitting at some point, where, as the train loss decreases, the test loss is increasing. Whereas, for PINN models we observe a converging training loss, where in multiple cases, test loss continues decreasing (e.g. SID#6).

Supplementary Figure 12

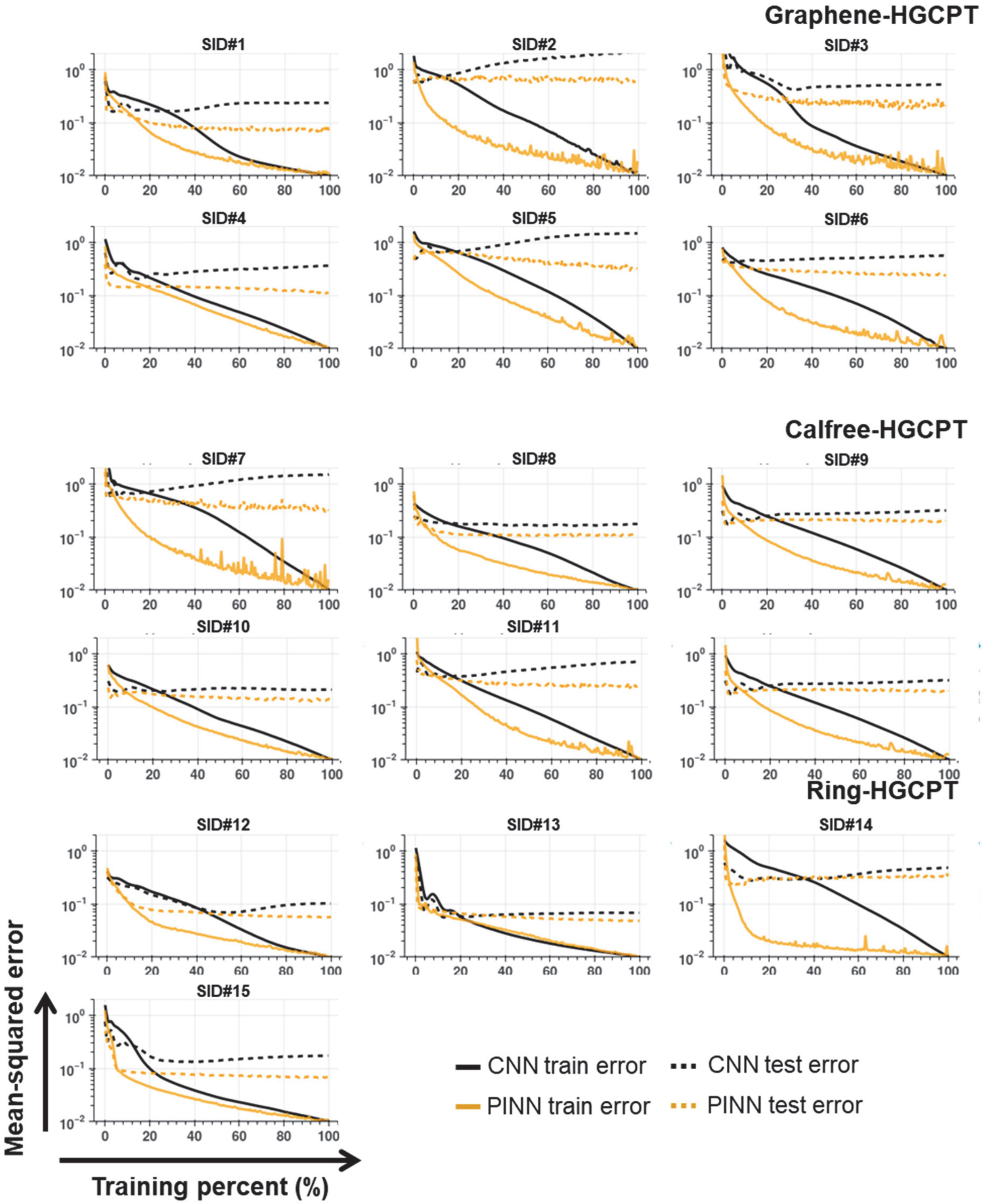

Supplementary Figure 12. PINN and CNN training and testing error progression with training for PP estimation. For the training errors, only the supervised training losses (i.e., mean-squared different between prediction and true) are shown. The trainings are stopped when the supervised training errors reached 0.01. We observe that the CNN models in almost all cases begin overfitting at some point, where, as the train loss decreases, the test loss is increasing. Whereas, for PINN models we observe a converging training loss, where in multiple cases, test loss continues decreasing (e.g. SID#5).

Supplementary Figure 13

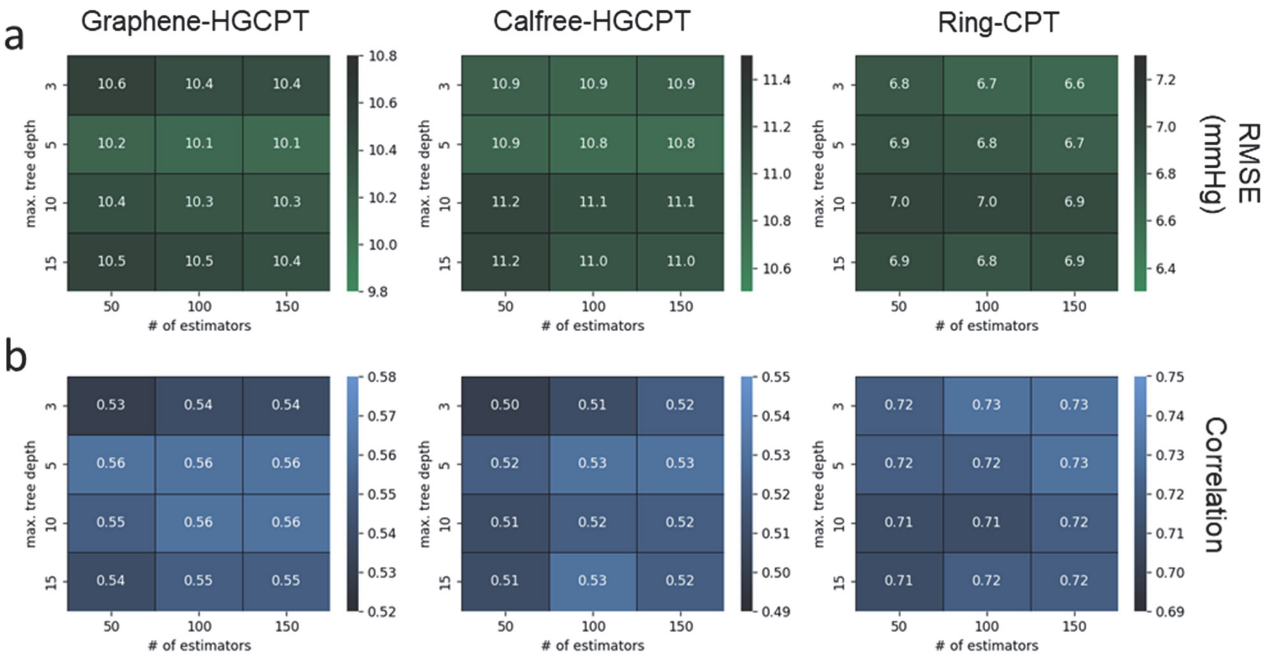

Supplementary Figure 13. AdaBoost regressor hyperparameter grid search. We use Decision Tree regressor as the base estimator. Two main hyperparameters are assessed. Max. tree depth (3, 5, 10, or 15): The maximum depth of the Decision Tree. # of estimators (50, 100, 150): the maximum number of estimators at which boosting is terminated. The model is trained with SBP data, under 4-folds cross-validation train-test split. The analysis is presented separately for Graphene-HGCPT (left plots), Calfree-HGCPT (middle plots), Ring-CPT (right plots) datasets. **a.** Root-mean-squared errors (RMSE) in estimating SBP based on different hyper-parameter settings. **b.** Correlation coefficient calculated between estimated and true SBP values. The changes in performances across different hyperparameter selections are marginal, since AdaBoost being less prone to overfitting due to the boosted learning, with the input parameters prevented from being jointly optimized.

Supplementary Note 1

Blood pressure (BP) is a frequently used cardiovascular (CV) parameter by clinicians to assess the cardiac and circulatory health and associated risk factors and disorders. The conventional BP measurement is based on an oscillometric cuff inflation/deflation, capturing single systolic (SBP), diastolic (DPB) and pulse pressure (PP) values. This results in infrequent readings of BP, and therefore can lead to misdiagnosis caused by inaccurate readings in the presence of measurement biases (e.g., *white coat syndrome*: an artificial rise in BP in medical clinics, while normal at home, *masked hypertension*: BP recorded at normal ranges in clinics, while being high in ambulatory settings)[13]. Moreover, the inflating cuff causes discomfort, preventing the continuous and frequent use to capture useful trends and levels (e.g., normotensive, hypertensive, hypotensive) in BP in ambulatory and nocturnal settings that are necessary to provide accurate CV health assessment and management[14–16]. State-of-the-art wearable cuffless BP technologies address the shortcomings of the cuff, with a trade-off in the fidelity. These technologies leverage non-invasive and unobtrusive modalities (e.g., PPG[17], bioimpedance[2], capacitive[18], ultrasound[19]) to capture certain physiological events and parameters that are relevant to BP. Bioimpedance particularly provides unique advantages over other modalities when used for measuring blood volume changes as the BP pulse wave travels through arteries. These are: (i) due to the use of high-frequency electric current penetration into deep-tissue, bioimpedance sensors capture changes at artery levels rather than skin/capillary level, unlike optical modalities limited with skin-level light penetration[20]; (ii) bioimpedance is insensitive to the variations in participants' skin tone and body-mass index (BMI), unlike optical modalities, where light is attenuated before reaching the arteries for individuals with darker skin tones and higher BMI[20–22]; (iii) bioimpedance provides an area of sensing with proper placement of the skin contact electrodes[23,24], unlike ultrasound[19,25] and radar/Wi-Fi that are highly directional[26].

Supplementary Note 2

Replacing the conventional cuff technology with an unobtrusive wearable sensor for blood pressure (BP) measurements can enable continuous BP monitoring in ambulatory and nocturnal settings. These measurements can address the challenges with infrequent cuff-based BP monitoring, where these challenges include measurement biases such as *white coat syndrome* – an artificial rise in BP in medical clinics, while normal at home, *masked hypertension* – BP recorded at normal ranges in clinics, while being high in ambulatory settings[13], and the inability to measure various trends and changes in BP especially during night time (e.g., nocturnal non-dipping BP[27]). In addition, continues access to BP for masses can generate hypertension awareness and lead to better diagnostic and management of related cardiovascular disorders. There are a rich variety of research-level and matured technologies investigated for cuffless BP monitoring. However, certain limitations should still be addressed for these technologies to become clinically viable.

The research-grade technologies investigate the use of novel materials and modalities that can non-invasively monitor certain hemodynamic parameters that is related to BP through the cardiovascular dynamics. Examples include ultrasound sensors[28,29], tonometers (pressure sensors)[30], ballistocardiographs (BCG)[31–33], and bioimpedance[2,7]. The ultrasound measures arterial blood volume and velocity changes via Doppler principles[19,34]. However, sensors placement is relatively difficult, generally requiring an operator interaction, hindering its wearable operation[35]. Tonometry technique involves a pressure sensor capturing the skin induced force due to the presence of BP[30,36,37]. However, it requires applanation of the arterial lumen via a cuff-like device adding inconvenience[30,38]. BCG measures the body movements due to BP wave, via accelerometers placed on bed-like tables or highly sensitive scales. While this method provides useful measurements, it is susceptible to motion artifacts[39]. Bioimpedance particularly provides unique advantages over other modalities when used for measuring blood volume changes. These are: (i) due to the use of high-frequency electric current penetration into deep-tissue, bioimpedance sensors capture changes at artery levels rather than skin/capillary level, unlike optical modalities limited with skin-level light penetration[20]; (ii) bioimpedance is insensitive to the variations in participants' skin tone and body-mass index (BMI), unlike optical modalities, where light is attenuated before reaching the arteries for individuals with darker skin tones and higher BMI[20–22]; (iii) bioimpedance provides an area of sensing with proper placement of the skin contact electrodes[23,24], unlike ultrasound[19,25] and radar/Wi-Fi that are highly directional[26].

There is a plurality of matured investigations focusing on capturing features with wearables that is related to hemodynamics and extracting BP based on its relationship with these hemodynamic features. Although, many commercially available devices came out of these investigations, they are not currently recommended for clinical applications[40,41]. These studies build on mainly pulse transit time (PTT) and pulse wave analysis (PWA)[39,41–44]. PTT is defined as the time delay between proximal and distal arterial waveforms, where it is inversely related to SBP and DBP, due to the compliant artery characteristics[42,43]. To measure PTT, a common technique is to use an ECG in conjunction with an optical photoplethysmography (PPG) sensor at the wrist or finger tips. ECG provides the electrical activation of the heart, marking the start of the pulse travel, while PPG captures the pulse arrival to the distal sensor. This approach requires the use of multiple sensors, instigating inconvenience for users. On the other hand, PWA allows for a measurement conducted from a single sensor, where the unique arterial waveform is analyzed to capture expert-designed features that are related to BP[45,46]. Together with machine learning, PWA is gaining popularity as the signals can be conveniently in wearable form-factors and can be related to BP[47–49]. However, both PTT and PWA-based cuffless BP monitoring approaches rely on generalized expert-feature and BP relationships that may not exhibit consistent behavior across individuals, and the arterial waveform shows dynamic intra-subject changes due to aging and arterial stiffening, and other underlying factors that alter the arterial dynamics, which are not properly formulized[45,50,51]. Therefore, the machine learning algorithms mapping features to BP need to be trained on large datasets to account for inter-subject variabilities and calibrated frequently for intra-subject changes. We believe the use of PINNs can address these challenges as it exploits the cardiovascular dynamics behind the mapping of expert features and BP. In addition, we show that, when minimal amounts of ground truth information are used during neural network training, PINNs overperform against all tested state-of-the-art deep learning models that do not require features used for BP estimation.

Although, there are many published studies on calibration paradigms for cuffless BP monitoring, there are certain issues in the presentation of the performance with potentially inconclusive findings as pointed out by several recent reviews on the topic[40,51–53]. A fundamental challenge is the missing analysis on datasets with inter- and intra-individual BP variations. Our study is conducted on a dataset that includes certain interventions that invoke BP changes (cold pressor test, hand-gripper exercise). Supplementary Table 1 shows the inter- and intra-individual BP ranges and the percentage of data points within each BP category (normal, elevated, stage 1, stage 2, and hypertension crisis). In addition, we use Finapres NOVA finger cuff to obtain numerous reference cuff BP measurements from each individual, that is otherwise impractical with ambulatory cuff-based BP monitors. Another limitation with certain cuffless BP studies is their use of demographics (e.g., age, sex) as additional features in their machine learning algorithms[40,54,55]. This leads to less realistic assessment of the device performance, given the known correlation of demographics with BP[40,41]. In our study, we only use raw bioimpedance waveforms and extract physiological features, and do not take individual demographic information as an input.

Supplementary Note 3

PINN evaluation under inter-scenario and inter-subject strategies. In the first analysis, we divide the dataset into six bins based on the equal BP ranges, where we use the two bins corresponding to the lowest and highest BP ranges for training, and the remaining four bins for testing. For example, if a participant's SBP is ranging from 120 mmHg to 180 mmHg, we use the BP ranges of 120-130 mmHg and 170-180 mmHg for training, whereas the samples within the BP range of 130-170 are used for testing. The intuition behind this evaluation strategy is to test the performance of the AI models that are trained on low and high extrema values of the dataset and tested on a range of values that have not been seen by the models before. We apply the same train/test condition to the conventional neural network model (referred to as CNN in the manuscript), as well as to the three machine learning models (AdaBoost, Rocket, and Random Forest regression models). The results of this analysis are shared in Supplementary Table 17, with values averaged over all participants (N=15). We observe that the proposed PINNs overperform all other learning models, achieving -1.3 ± 7.0 mmHg and -0.7 ± 5.8 mmHg estimation accuracies, corresponding to 1.5 and 1.4 times lower standard deviation of errors than the best performance obtained with the state-of-the-art-models, for SBP and DBP respectively. The superior performance of PINN is due to the physics-based constrained on the model predictions based on the approximated input-output relationships.

In the second analysis, we evaluate the models in inter-subject settings (e.g., leave-one-subject-out training), where we train the models based on the data from all subjects except one (N=14), and test on the excluded subject (N=1), with four-point calibration. For this analysis;

- The training data points for each subject are selected based on the minimal training criterion to be cognizant of the infeasibility of retaining beat-to-beat BP ground truth measurements (such as with highly sophisticated devices like Finapres NOVA) for most applications even for training purposes.
- Four points from the test subject's labeled BP data are selected for model calibration purposes. These are; the first and last BP points, and maximum and minimum BP points. The intuition behind this selection is the feasibility of obtaining the first and last BP points for each individual with a conventional cuff-based BP, where the maximum and minimum BP points can be accessed based on an immediate cuff-based BP measurement followed by a specific maneuver (e.g., cold-pressor exercise for maximum BP, Valsalva maneuver for minimum BP).
- Given that the classical machine learning models (e.g., AdaBoost, Rocket, and Random Forest regression models) cannot be retrained, to enable proper calibration with these models, we emphasize the first calibration point (i.e., start) of the test subject's data by providing the model training and estimation opportunity over the changes of BP from the first point both in training and testing datasets (due to the availability of first calibration point). Once the training is finalized we add the starting BP value to the model predictions to get the final model BP estimations. This change-estimation modification in the datasets is applied to all models - including the DNN models - to obtain a fair comparison.
- For CNN and PINN models, an initial DNN model having the same architecture is trained on the training dataset with a conventional loss function, where 20-percent of this training data is used for validation. The model weights yielding the lowest validation loss are selected to obtain the most generalized solution. This DNN model is used for the starting point for CNN and PINN models, where both models are calibrated with the three points (i.e., start, maximum and minimum) of the four calibration points. The fourth calibration point (i.e., end) is used for validation. The only difference between CNN and PINN models is the physics-based loss function calculated over the test subject's data and used for PINN model optimization. For both models, the weights yielding the lowest validation loss are selected to generate final predictions.
- The classical machine learning models are trained on the dataset that combines the training and calibration points, due to the infeasibility of machine learning model re-training that is otherwise possible with DNN models.

Supplementary Figures 8 and 9 show the Bland-Altman and Pearson's correlation analyses for CNN, AdaBoost Regressor, Rocket Regressor, and Random Forest Regressor models in comparison with the PINN models for SBP and DBP, respectively. Supplementary Table 18 shows the results averaged over all participants. We observe that PINNs provide significant improvements against the state-of-the-art models, achieving 0.2 ± 12.1 mmHg and 1.6 ± 8.7 mmHg estimation accuracies (1.3 and 1.1 times lower standard deviation of errors compared to the best performance obtained with the state-of-the-art-models), for SBP and DBP, respectively.

Supplementary References

1. Whelton, P. K. *et al.* 2017 ACC/AHA/AAPA/ABC/ACPM/AGS/APhA/ASH/ASPC/NMA/PCNA guideline for the prevention, detection, evaluation, and management of high blood pressure in adults: a report of the American College of Cardiology/American Heart Association Task Force on Clinical Pr. *J. Am. Coll. Cardiol.* **71**, e127--e248 (2018).
2. Kireev, D. *et al.* Continuous cuffless monitoring of arterial blood pressure via graphene bioimpedance tattoos. *Nat. Nanotechnol.* 1–7 (2022).
3. Ibrahim, B. & Jafari, R. Cuffless blood pressure monitoring from a wristband with calibration-free algorithms for sensing location based on bio-impedance sensor array and autoencoder. *Sci. Rep.* **12**, 1–14 (2022).
4. Sel, K. *et al.* Continuous cuffless blood pressure monitoring with a wearable ring bioimpedance device. *npj Digit. Med.* **6**, 59 (2023).
5. Klabunde, R. E. Arterial and aortic pulse pressure. *Image for Cardiovascular Physiology Concepts, Richard E Klabunde PhD* at https://www.cvphysiology.com/Blood Pressure/BP003.
6. Messas, E., Pernot, M. & Couade, M. Arterial wall elasticity: state of the art and future prospects. *Diagn. Interv. Imaging* **94**, 561–569 (2013).
7. Huynh, T. H., Jafari, R. & Chung, W.-Y. An accurate bioimpedance measurement system for blood pressure monitoring. *Sensors* **18**, 2095 (2018).
8. Tang, X., Jankovic, M. & Jafari, R. A Non-invasive Radial Arterial Compliance Measuring Method using Bio-Impedance. in *2021 43rd Annual International Conference of the IEEE Engineering in Medicine & Biology Society (EMBC)* 2330–2334 (2021).
9. Brillante, D. G., O'sullivan, A. J. & Howes, L. G. Arterial stiffness indices in healthy volunteers using non-invasive digital photoplethysmography. *Blood Press.* **17**, 116–123 (2008).
10. Mourot, L., Bouhaddi, M. & Regnard, J. Effects of the cold pressor test on cardiac autonomic control in normal subjects. *Physiol. Res.* **58**, (2009).
11. Schwabe, L. & Schächinger, H. Ten years of research with the Socially Evaluated Cold Pressor Test: Data from the past and guidelines for the future. *Psychoneuroendocrinology* **92**, 155–161 (2018).
12. Gourine, A. V & Ackland, G. L. Cardiac vagus and exercise. *Physiology* **34**, 71–80 (2019).
13. Mancia, G., Bombelli, M., Seravalle, G. & Grassi, G. Diagnosis and management of patients with white-coat and masked hypertension. *Nat. Rev. Cardiol.* **8**, 686–693 (2011).
14. Flint, A. C. *et al.* Effect of systolic and diastolic blood pressure on cardiovascular outcomes. *N. Engl. J. Med.* **381**, 243–251 (2019).
15. Kario, K. Management of hypertension in the digital era: small wearable monitoring devices for remote blood pressure monitoring. *Hypertension* **76**, 640–650 (2020).
16. Carey, R. M., Muntner, P., Bosworth, H. B. & Whelton, P. K. Prevention and control of hypertension: JACC health promotion series. *J. Am. Coll. Cardiol.* **72**, 1278–1293 (2018).
17. Shin, H. & Min, S. D. Feasibility study for the non-invasive blood pressure estimation based on ppg morphology: Normotensive subject study. *Biomed. Eng. Online* **16**, 1–14 (2017).
18. Kim, J. *et al.* Soft wearable pressure sensors for beat-to-beat blood pressure monitoring. *Adv. Healthc. Mater.* **8**, 1900109 (2019).
19. Soleimani, E., Mokhtari-Dizaji, M., Fatouraee, N. & Saberi, H. Assessing the blood pressure waveform of the carotid artery using an ultrasound image processing method. *Ultrasonography* **36**, 144 (2017).
20. Bent, B., Goldstein, B. A., Kibbe, W. & Dunn, J. P. Investigating sources of inaccuracy in wearable optical heart rate sensors. *NPJ Digit. Med.* **3**, (2020).
21. Fine, J. *et al.* Sources of Inaccuracy in Photoplethysmography for Continuous Cardiovascular Monitoring. *Biosensors* **11**, (2021).
22. Boonya-ananta, T., Rodriguez, A. J., Hansen, A. K., Hutcheson, J. D. & Ramella-Roman, J. C. Monte Carlo Modeling of a Photoplethysmographic (PPG) in Individuals with Obesity. in *Biophotonics Congress: Biomedical Optics 2020 (Translational, Microscopy, OCT, OTS, BRAIN)* JTu3A.39 (Optica Publishing Group, 2020).
23. Sel, K., Osman, D. & Jafari, R. Non-invasive cardiac and respiratory activity assessment from various human body locations using bioimpedance. *IEEE open J. Eng. Med. Biol.* **2**, 210–217 (2021).
24. Sel, K., Ibrahim, B. & Jafari, R. ImpediBands: Body Coupled Bio-Impedance Patches for Physiological Sensing Proof of Concept. *IEEE Trans. Biomed. Circuits Syst.* **14**, 757–774 (2020).
25. Wang, C. *et al.* Bioadhesive ultrasound for long-term continuous imaging of diverse organs. *Science (80-.).* **377**, 517–523 (2022).
26. Li, X., Qiao, D., Li, Y. & Dai, H. A novel through-wall respiration detection algorithm using uwb radar. in *Engineering in Medicine and Biology Society (EMBC), 2013 35th Annual International Conference of the IEEE* 1013–1016 (2013).
27. Cho, S. M. J. *et al.* Association between nocturnal blood pressure dipping and chronic kidney disease among patients with controlled office blood pressure. *Am. J. Hypertens.* **34**, 821–830 (2021).
28. Wang, C. *et al.* Continuous monitoring of deep-tissue haemodynamics with stretchable ultrasonic phased arrays. *Nat. Biomed. Eng.* **5**, (2021).
29. Wang, C. *et al.* Monitoring of the central blood pressure waveform via a conformal ultrasonic device. *Nat. Biomed. Eng.* **2**, 687–695 (2018).
30. Mieloszyk, R. *et al.* A comparison of wearable tonometry, photoplethysmography, and electrocardiography for cuffless measurement of blood pressure in an ambulatory setting. *IEEE J. Biomed. Heal. Informatics* **26**, 2864–2875 (2022).
31. Inan, O. T. *et al.* Ballistocardiography and seismocardiography: A review of recent advances. *IEEE J. Biomed. Heal. informatics* **19**, 1414–1427 (2014).
32. Kim, C.-S., Carek, A. M., Inan, O. T., Mukkamala, R. & Hahn, J.-O. Ballistocardiogram-based approach to cuffless blood pressure monitoring: Proof of concept and potential challenges. *IEEE Trans. Biomed. Eng.* **65**, 2384–2391 (2018).
33. Kim, C.-S. *et al.* Ballistocardiogram: Mechanism and potential for unobtrusive cardiovascular health monitoring. *Sci. Rep.* **6**, 1–6 (2016).

34. Gill, R. W. Measurement of blood flow by ultrasound: accuracy and sources of error. *Ultrasound Med. Biol.* **11**, 625–641 (1985).
35. Vappou, J., Luo, J., Okajima, K., Di Tullio, M. & Konofagou, E. E. Non-invasive measurement of local pulse pressure by pulse wave-based ultrasound manometry (PWUM). *Physiol. Meas.* **32**, 1653 (2011).
36. Drzewiecki, G. M., Melbin, J. & Noordergraaf, A. Arterial tonometry: review and analysis. *J. Biomech.* **16**, 141–152 (1983).
37. Kemmotsu, O. *et al.* Blood pressure measurement by arterial tonometry in controlled hypotension. *Anesth. Analg.* **73**, 54–58 (1991).
38. Athaya, T. & Choi, S. A review of noninvasive methodologies to estimate the blood pressure waveform. *Sensors* **22**, 3953 (2022).
39. Mukkamala, R., Stergiou, G. S. & Avolio, A. P. Cuffless blood pressure measurement. *Annu. Rev. Biomed. Eng.* **24**, 203–230 (2022).
40. Mukkamala, R. *et al.* Evaluation of the accuracy of cuffless blood pressure measurement devices: challenges and proposals. *Hypertension* **78**, 1161–1167 (2021).
41. Stergiou, G. S. *et al.* Cuffless blood pressure measuring devices: review and statement by the European Society of Hypertension Working Group on Blood Pressure Monitoring and Cardiovascular Variability. *J. Hypertens.* **40**, 1449–1460 (2022).
42. Ding, X. & Zhang, Y.-T. Pulse transit time technique for cuffless unobtrusive blood pressure measurement: from theory to algorithm. *Biomed. Eng. Lett.* **9**, 37–52 (2019).
43. Barvik, D., Cerny, M., Penhaker, M. & Noury, N. Noninvasive Continuous Blood Pressure Estimation from Pulse Transit Time: A review of the calibration models. *IEEE Rev. Biomed. Eng.* (2021).
44. Mukkamala, R. *et al.* Toward ubiquitous blood pressure monitoring via pulse transit time: theory and practice. *IEEE Trans. Biomed. Eng.* **62**, 1879–1901 (2015).
45. Natarajan, K. *et al.* Photoplethysmography fast upstroke time intervals can be useful features for cuff-less measurement of blood pressure changes in humans. *IEEE Trans. Biomed. Eng.* **69**, 53–62 (2021).
46. Hofmann, G. *et al.* A novel smartphone app for blood pressure measurement: a proof-of-concept study against an arterial catheter. *J. Clin. Monit. Comput.* 1–11 (2022).
47. El-Hajj, C. & Kyriacou, P. A. A review of machine learning techniques in photoplethysmography for the non-invasive cuff-less measurement of blood pressure. *Biomed. Signal Process. Control* **58**, 101870 (2020).
48. Solà, J. & Delgado-Gonzalo, R. The Handbook of Cuffless Blood Pressure Monitoring. *Cham Springer* (2019).
49. Agham, N. D. & Chaskar, U. M. Learning and non-learning algorithms for cuffless blood pressure measurement: a review. *Med. Biol. Eng. Comput.* **59**, 1201–1222 (2021).
50. Allen, J. & Murray, A. Age-related changes in the characteristics of the photoplethysmographic pulse shape at various body sites. *Physiol. Meas.* **24**, 297 (2003).
51. Avolio, A. *et al.* Challenges presented by cuffless measurement of blood pressure if adopted for diagnosis and treatment of hypertension. *Pulse* **10**, 34–45 (2022).
52. Picone, D. S. *et al.* Nonvalidated home blood pressure devices dominate the online marketplace in Australia: major implications for cardiovascular risk management. *Hypertension* **75**, 1593–1599 (2020).
53. Stergiou, G. S. *et al.* 2021 European Society of Hypertension practice guidelines for office and out-of-office blood pressure measurement. *J. Hypertens.* **39**, 1293–1302 (2021).
54. Chowdhury, M. H. *et al.* Estimating blood pressure from the photoplethysmogram signal and demographic features using machine learning techniques. *Sensors* **20**, 3127 (2020).
55. Yao, P. *et al.* Multi-Dimensional Feature Combination Method for Continuous Blood Pressure Measurement Based on Wrist PPG Sensor. *IEEE J. Biomed. Heal. Informatics* **26**, 3708–3719 (2022).
